# Bed Bugs (Hemiptera, Cimicidae): A Global Challenge for Public Health and Control Management

**DOI:** 10.3390/diagnostics13132281

**Published:** 2023-07-05

**Authors:** Mohammad Akhoundi, Coralie Zumelzu, Denis Sereno, Anthony Marteau, Sophie Brun, Julie Jan, Arezki Izri

**Affiliations:** 1 Parasitology-Mycology Department, Avicenne Hospital, AP-HP, Sorbonne Paris Nord University, 93000 Bobigny, France; 2 Dermatology Department, AP-HP, Avicenne Hospital, 93000 Bobigny, France; 3 Parasite Infectiology and Public Health Research Group, Institute de Recherche Pour le Développement, InterTryp, Montpellier University, 34032 Montpellier, France; 4 Agence Régionale de Santé (ARS), Île-de-France, 75935 Paris, France; 5 Unité des Virus Émergents (UVE: Aix-Marseille Univ-IRD 190-Inserm 1207-IHU Méditerranée Infection), 13005 Marseille, France

**Keywords:** bed bugs, clinical manifestations, diagnosis, treatment, control management

## Abstract

Bed bugs, *Cimex lectularius*, and *C. hemipterus* are among the most common ectoparasites in human life worldwide. They feed on humans of all ages and sexes across all socioeconomic levels. Bed bugs’ blood feeding is responsible for a wide range of clinical manifestations varying from minor reactions to bullous eruptions or severe allergies. In addition, they are responsible for considerable psychological distress. Therefore, diagnosis of bed bug bites and their consequence manifestations is beneficial in adapting remedies and treatment protocols advised by clinicians. So far, there is regrettably no definitive way to control these ectoparasites despite extensive efforts of public health authorities to manage them. An overview of the literature and medical documents gathered from bed bug-infested patients referred to the Parasitology and Dermatology departments of Avicenne Hospital (Bobigny, France) allowed us to document and illustrate a range of clinical disorders and psychological concerns caused by bed bugs’ bites and their clinical diagnosis. We also review the available tools currently used to control the bed bugs and present potential candidate methods for their successful eradication.

## 1. Introduction

Bed bugs are parasitic insects that feed on human blood. They have a long history of drastic presence in human communities with extended geographical dispersion worldwide. For many years, they have been a significant public health issue and probably one of the most common ectoparasites in human life [1]. Bed bugs belong to the Cimicidae family and the *Cimex* genus. The latter consists of 23 species feeding on birds, bats, and humans [2,3]. Some species occasionally feed on humans in the absence of their primary host. Bed bugs have five developmental life stages, from egg to adult. The females lay about five eggs daily (200–500 eggs in a lifetime). Eggs hatch within 4–12 days into the first instar nymph. Each nymphal stage takes a blood meal before molting to the next step [4]. All stages in both sexes feed exclusively on blood. In the adult stage, they are oval-shaped with flattened bodies and are approximately 5 mm long. Adults are reddish-brown, whereas the immature stages are smaller with light yellow color. According to species, the adults can live 6–12 months and may survive for an extended period without feeding (from 80 to 140 days) [4].

The two main bed bug species usually implicated in human infestations are *Cimex lectularius* and *C. hemipterus*. *Cimex lectularius*, a common bed bug, inhabit temperate regions of the Nearctic and Palearctic areas (Asia, Australia, Africa, and South America), whereas *C. hemipterus* is prevalent and familiar in tropical and subtropical regions [3,5]. Recently, both species have been found to migrate outside their traditional geographic areas. Besides tropical regions, *C. hemipterus* has recently expanded to other temperate zones in the Middle East, North Australia, the United States, Russia, Sweden, Italy, and France [6,7,8,9,10,11,12]. Similarly, *C. lectularius* has lately been reported from Madagascar [13].

The increase in international travel, immigration, and secondhand business has disrupted this classic division and extended their geographical dispersions, resulting in the sympatric occurrence of *C. lectularius* and *C. hemipterus*. In three recent decades, bed bugs’ infestation of human habitats has drastically increased, leading to a rise in bed bug concerns [14]. They are responsible for several clinical and psychological disorders [15,16]. In addition, they cause multiple economic problems affecting cultural and tourism industries (e.g., the economic impact of the resurgence was 100 million AUS dollars in Australia) [17]. During the coronavirus disease pandemic, there has been an increase in elderly neglect. Therefore, the need for social isolation during pandemics can contribute to elder abuse and neglect [18]. Nowadays, bed bug infestations are no longer limited to homes and hotels, and infestations have become common in private or public settings, including elder residences, healthcare facilities, and public transportation [19,20]. Bed bugs commonly live in diverse microhabitats close to humans, such as bed cracks, curtains, floorboards, wallpapers, upholstery fabrics, picture frames, electrical outlets, mattresses, carpets, or baseboards. They can spread by active or passive dispersal. Active dispersal occurs when a bed bug walks from one site to another location. A bed bug may spread between floors and rooms in hotels or apartment buildings. Passive dispersal occurs when bed bug travel by infested objects displacement, such as clothing, luggage, or furniture [4]. Based on the literature, there are several case reports in which the patients were infested in hotels, hospitals, dormitories, public transport, sheep, etc., and presented a variety of clinical manifestations, sometimes very complicated [21,22,23,24].

In this study, we overview current knowledge on the bed bugs and summarize the research findings on public health implications and control management of bed bug infestations. For this purpose, a narrative literature review was performed including research articles, books, and dissertations according to the PRISMA (Preferred Reporting Items for Systematic Reviews and Meta-Analyses) guideline [25]. The searches were performed in Scopus, PubMed, Science Direct, ProQuest, Web of Science, Springer, MEDLINE, and Google Scholar in five languages (English, French, German, Portuguese, and Spanish) without restriction by publication date. Relevant articles that met the mentioned criteria were selected. Duplicated articles and those with unrelated topics were excluded. A total of 128 articles were gathered. Among them, 89 met the study criteria were selected for review purposes.

## 2. Vectorial Transmission

Bed bugs harbor more than 40 infectious agents [15,26]. They include bacteria (such as *Borrelia recurrentis*, *B. duttoni*, *Coxiella burnetii*, and *Rickettsia rickettsii*) [27,28], fungi (e.g., *Aspergillus* spp.) [29], viruses (e.g., hepatitis B and HIV) [15], or parasites [15]. The first evidence of experimental infection of *C. lectularius* by *Trypanosoma cruzi* was reported by Blacklock in 1914 [30]. The competence of *C. lectularius* to act as a biological vector of *T. cruzi* (responsible agent of Chagas disease) and *Bartonella quintana* (causative agent of trench fever) has been recently confirmed in the laboratory [31,32]. In addition, several disease agents have been detected in the feces of bed bugs, such as *T. cruzi* [33], *Bacillus anthracis* [34], *Francisella tularensis* [35], *Brucella melitensis*, *B. abortus*, and *B. suis* [36], *Salmonella paratyphi* [37] as well as Yellow fever [38], Smallpox [39] and Lymphocytic choriomeningitis viruses [40]. Nevertheless, no evidence concerning the role of *Cimex* species in transmitting human pathogenic agents is available in endemic areas.

## 3. Clinical and Psychological Issues

**Figure 1:**
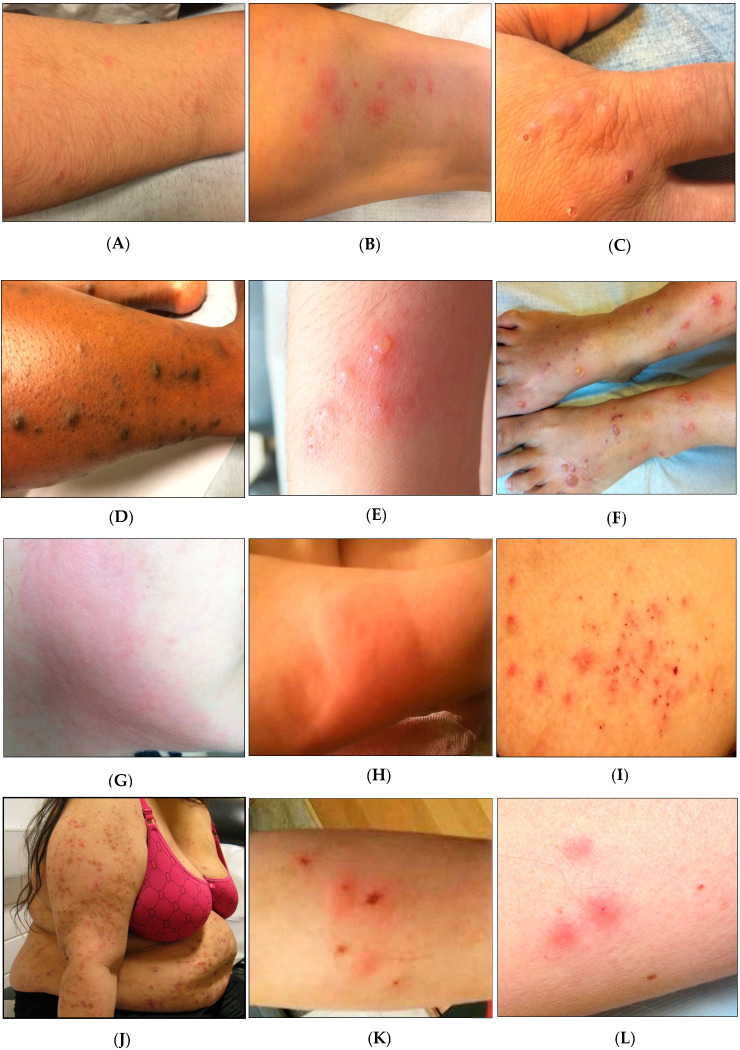
A wide range of clinical manifestations caused by bed bug bites (in the patients referred to the Parasitology Department of Avicenne Hospital, Bobigny, France). (**A**) Minor reaction. (**B**) Maculopapule. (**C**) Papule. (**D**) Nodule. (**E**) Vesicle. (**F**) Bullae. (**G**) Erythema. (**H**) Edema. (**I**) Eczematiform lesion. (**J**) Generalized lesion. (**K**) Secondary infection. (**L**) Bite spot of bed bug.

Blood feeding of bed bugs can cause an array of adverse health effects in humans [15,22]. The reactions to bed bug bite commonly form due to an immunologic response to saliva proteins. Forty-six proteins have previously been identified in *C. lectularius* salivary glands, with Nitrophorin and apyrase as the most common ones [41]. They are responsible for multiple biological functions, including preventing host clot formation, inhibiting platelet aggregation, and promoting vasodilation; some may act as antimicrobial agents [42]. The clinical reactions to bed bug bites include cases with no reaction (10 to 30% of cases) [43], minor reaction or bump (Figure 1A), maculopapule (Figure 1B), papule (Figure 1C), nodule (Figure 1D), vesicle (Figure 1E), bullae (Figure 1F), erythema (Figure 1G), edema (Figure 1H), eczematiform lesion (Figure 1I), scratching lesion (Figure 1J), and secondary infection (Figure 1K) [15]. Although the bite spot of bed bugs cannot be clearly visible, it can occasionally appear with a tiny black spot on the bite site (Figure 1L). Because bed bugs feed at night and inject an anesthetic when biting, the first bite is not commonly felt, and most patients do not exhibit clinical reactions. Patients over 65 and children between 1 and 10 years of age reported lower reaction rates (58% and 59%, respectively) [43]. Among mentioned clinical manifestations, maculopapular and erythematous lesions are usually the most common clinical reactions to bed bug bites. After biting, cutaneous symptoms often appear immediately. In repeated bites, some individuals may have enlarged pruritus [44,45]. After persistent scratching, secondary bacterial or fungal infection of lesions may occur [46,47,48] (Figure 1I). Other clinical disorders might happen in rare cases, such as asthmatic reactions, urticaria, and anaphylaxis [49,50].

Besides clinical issues, bed bugs can cause psychological disorders resulting from their presence in an infested location. Unfortunately, the psychological consequences of bed bug bites are underestimated, leading to little knowledge of these aspects of the disorder. Bed bugs infestation can cause significant psychological disorders such as anxiety, nightmares, phobia, hypervigilance, insomnia, poor self-esteem, significant adverse effect on the quality of life, personal dysfunction, and a substantial socioeconomic burden to society [51,52]. Therefore, bed bugs are considered a source of stress and severe psychiatric consequences for those susceptible [51].

## 4. Diagnosis

**Figure 2:**
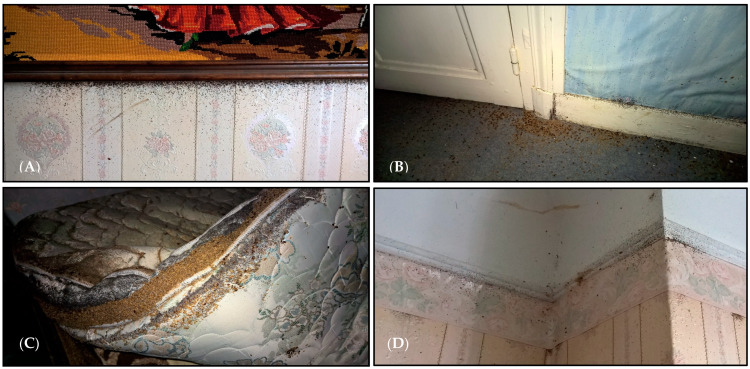
Bed bug infestations in various parts of an apartment in Paris (inspected in this study). (**A**) Numerous excreted black spots of bed bug blood digestion behind the painting panel, (**B**) Multiple living bed bugs with different life stages hidden in the baseboards, (**C**) Huge quantity of exoskeletons and living bed bugs reproduced in the bed mattress, (**D**) Bed bugs colonized behind the wall sheets.

Bed bugs are frequently found in beds, mattresses, box springs, wall cracks, crevices, electrical outlets, or wooden furniture, which can constitute microhabitats where bed bugs can hide to stay close to humans. Infestations are common in private or social dwellings, hospitals, hotels, tourist residences, or public transport (Figure 2). The infestation can be detected during a visual inspection according to several criteria, mainly the bite appearance, infestation signs, and history. The infestation scale can be categorized from 0 (no infestation) to 5 (hyperinfestation), depending on the specific criteria (Table 1).

Bed bugs feed nocturnally on exposed body areas like arms, legs, face, and neck. Their biting is painless that can happen throughout the year. They are attracted to their hosts primarily by carbon dioxide, secondarily by warmth, and also by certain chemicals. After the initial bite, cutaneous symptoms can appear within a few minutes to days. Bed bug bites can cause several clinical manifestations similar to the bites of other hematophagous arthropods, like mosquitos or fleas.

**Figure 3:**
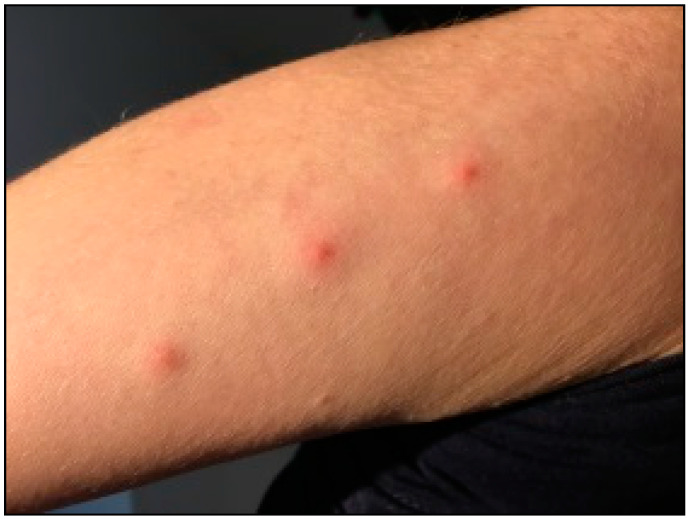
Strait pattern of bed bugs’ bite.

Nevertheless, some symptoms can discriminate bed bug bites from others ones. In the initial step, bed bug bites can be characterized by 3 to 5 red bumps commonly appearing in a straight line or zigzag pattern (Figure 3). Although some bites may occur sporadically, most of them happen in a row or cluster of at least three or more bites (namely “breakfast, lunch, and dinner”) [52].

Conversely, mosquito biting activity is sporadic and most commonly occurs in the evening or night (according to species) or at specific months or seasons. Bed bug infestation is usually looked for after a clinical diagnosis of bed bug bites. Similar symptoms in people sharing a bed, onset of the lesions after traveling or sleeping away from home, detection of bed bug fecal matter in or around the bed, or disappearance of symptoms after changing sleeping place should trigger suspicion of infestation.

Blood sampling is not generally recommended for a clinical examination, although in some allergic cases, moderate to high eosinophilia can be highlighted [53]. Although a biopsy is often unnecessary, it can differentiate between insect bites and lesions caused by other causative agents. In case of severe allergy, eosinophilia may be necessary. In rare cases, bed bugs can colonize human hosts [24], and their bite can also be considered a possible cause of chronic blood loss and anemia in heavily infested dwellings, particularly among elders [54]. This issue becomes more critical considering the increasing number of bed bug infestations worldwide and the aging in European countries. Psychiatric disorders (depression and dementia) and the individuals’ social isolation can worsen these conditions. This condition is commonly observed among elders who lose autonomy and live in poor conditions. The entomo-clinical discriminative characteristics of bed bug bites compared to other hematophagous arthropods’ bite is given in Table 2. Recognition of entomological characteristics, clinical presentations, diagnostic features, and differential diagnosis can support rapid identification of the patients exposed to bed bug infestations and their appropriate management [55].

## 5. Treatment

Symptoms following a bed bug bite are usually self-limited and can heal within one to two weeks without intervention. Therefore, bed bug bites are generally treated symptomatically. Washing the bite spots with soap and water is a frontline treatment preventing secondary skin infection and reducing itchiness [56]. Over-the-counter oral antihistamines like Benadryl may be beneficial in alleviating pruritus and swelling cases. Cold compress and topical/oral steroids are other remedies for relieving pruritus and reducing inflammation. Topical or oral antibiotics may be another option to treat secondary cutaneous bacterial infections [15]. In case of severe systemic allergic reactions, an injection of an antihistamine, corticosteroid, or epinephrine (adrenaline) can be recommended [57].

## 6. Current Challenge of Bed Bug Infestation and In-Access Management

The global resurgence of bed bugs has been and continues to be a public health concern and a significant challenge to pest management. Proper awareness and identification of bed bug infestations are essential to guide treatment and eradication. Bed bug control is expensive, amounting to billions of dollars each year globally [58]. Unfortunately, socially disadvantaged persons are the most impacted by bed bugs. Therefore, many infestations may go unreported or untreated, and the poorer sectors of society have become the reservoir for bed bugs [59].

Bed bugs are among the most challenging insects to control due to their resistance to different families of insecticides, the lack of other effective control methods, and the behavior of these insects that take advantage of the slightest hiding place in the human environment to hide and reproduce [43,60]. Despite significant developments in control strategies over the last three decades, they continue to invade, preferentially those from low socioeconomic communities, due to inaccessibility or limited effective control options [61]. Therefore, infestations in low-income houses can become massive and serve as insecticide-resistant bed bug reservoirs for spread throughout the community. However, with regard to the growing concerns reported worldwide (officially reported by 135 countries on five continents) [3], controlling these ectoparasites is inevitable. The longtime coevolution and prolonged presence of bed bugs in human dwellings have led to the development of numerous detection and monitoring tools and several control/eradication methods [62,63]. Nevertheless, there is still no absolute solution for their eradication.

Early detection of bed bugs is a prerequisite key factor in managing bed bug infestations, in reducing both the costs associated with bed bug management and the spread of bed bugs from infested dwellings to new locations. The methods may include surveys, visual inspections, canine detection, bed bug monitors, and traps. The query and interviewing residents about the history of bed bug infestations can be helpful. Bed bugs can be challenging to detect when their numbers are low. Visual inspection is the most common prerequisite screening method used by pest management professionals (PMPs) to detect bed bugs and ascertain the infestation level of an infested location, i.e., room, apartment, or building. It includes inspecting all wall cracks, bed and furniture crevices, electrical switch plates, electric cable channels, etc. In addition, the presence of hatched eggs, exoskeletons, or bed bug fecal spots on the mattress and bedding attests to the bed bugs’ presence in an infested location. However, the effectiveness of visual inspections relies on the experience and thoroughness of the inspector. Canine detection is another inspection tool that has been increasingly used in recent years [64]. However, this method reports diverse sensitivity rates ranging from 44 to 95% [65,66]. Volatile organic compounds (VOCs), semiochemicals in a gaseous phase with high vapor pressure, include a large group of various chemical compounds emitted by bed bugs. They have been proposed as a promising tool for bed bug detection [67]. Sampling and analyzing indoor air of an infested room using gas chromatography–mass spectrometry to characterize bed bugs’ VOCs demonstrated to be effective in identifying individual compounds in the blend of captured volatiles. This approach is more reliable than conventional detection methods, with no need for repeated inspections, household furniture moving, or resident rehousing for bed bugs’ VOC detection. On the other side, bed bug monitoring can be passive (without lures such as sticky pads for trapping) or active (with a lure such as heat or CO_2_). In the latter case, it requires the regular replacement of consumable lures, increasing charges and making them less feasible for routine usage, especially in low-income housing.

Integrated Pest Management (IPM) strategies against bed bug infestations are gathered in “Codes of practice” documents. They rely on two main sections: (i) chemical and (ii) non-chemical controls.

Insecticides have been the principal means to control bed bug infestations. The discovery and widespread use of organochlorine DDT in the 1940s led to a decline in bed bug populations for several decades, particularly in developed countries. In the 1950s and 1960s, organochlorine insecticides were replaced by organophosphate and carbamate. At the end of the 1980s and 1990s, pyrethroid insecticides such as permethrin, cypermethrin, and deltamethrin were introduced as alternatives. However, they have lesser residual stability than organochlorines and organophosphates [61,68]. Along wide use of mentioned insecticide, several resistance cases of *C. lectularius* and *C. hemipterus* populations were reported worldwide [69,70]. One of the first insecticide-resistant cases (DDT) was reported in 1947 in Hawaii [71]. Besides resistance to DDT, chemical control failures were also reported with other organochlorines (e.g., gamma-HCH, methoxychlor, dieldrin, aldrin, and endrin), organophosphates (e.g., malathion) and carbamates (e.g., carbaryl) from 1957 to 1972 in different regions of the world [61,72,73]. Furthermore, field-caught bed bugs were resistant to pyrethroids such as neopynamine and sumithrin [61,74]. In recent years, several cases of inefficient control with new-generation chemicals such as nicotinoides (e.g., dinotefuran, imidacloprid, thiamethoxam, and acetamiprid) have been reported worldwide [61,75].

Applying chemical insecticides to control hematophagous insects, including bed bugs, has resulted in the emergence of insecticide-resistant populations worldwide, making this control ineffective. In addition, they are toxic to human health and the environment leading to the spread of bed bugs to nearby non-infested locations. Repeated failure of the treatments due to chemical-insecticide resistance has led to the development of non-chemical controls as an alternative offering several advantages. It consists of many safe and effective options primarily based on mechanical, physical, and biological control tools and methodologies recommended to be applied on the same day.

The first step is to properly empty the infested location from textiles, e.g., bedding sheets, mattresses, clothes, blankets, etc., and to remove adult and immature bed bugs with a hand-held aspirator. Nevertheless, it does not kill them and does not remove their attached eggs as well. It effectively decreases the bed bugs population in an infested dwelling and helps eliminate some bed bugs before undertaking other treatment measures. Furthermore, vacuuming may also reduce allergens associated with a bed bug infestation. The aspiration bags must be closed or packed in a plastic bag to prevent infestation of neighbor locations [76,77]. Sealants are another recommended action to fill potential harborages on furniture and around rooms. Washing clothes, bedding, mattress, curtains, etc., at 60 °C is also effective in removing all life stages [78]. If an item cannot be laundered, placing it in a plastic bag is recommended to be appropriately treated with other more suitable methods such as heating or freezing. Bed bugs are sensitive to temperature extremes. Heating is a frequently used non-chemical control method to provide an alternative to insecticides. It is cost-effective, safe with no toxicity to humans, and eco-friendly. It has been used in various forms, including dry heating, by a specific machine, heated tents, or surface application by luggage [79,80]. Due to its low side effects, it is recommended for bed bug control, particularly for disinfecting objects or furniture. Heat treatment of a whole room with their belongings is an effective method currently used to control bed bugs [81]. The effectiveness of step-function 60 °C heat exposure was demonstrated in eliminating the adult bed bugs in any environmental condition (covered by tissues, furniture, mattress, or blanket) within 120 min.

Regarding no need for resident rehousing (moving before or during the treatment) and replacing the furniture, this practice represents an attractive method from the users’ point of view. Furthermore, reintegration into the treated dwelling is immediate at the end of the intervention, making this treatment economical. Moreover, bed bugs are less likely to develop heat resistance than to develop resistance to other treatment methods. Steam treatment is another effective method for killing all bed bugs’ developmental stages [82,83]. A recent study conducted by Chebbah et al. [84] reports cold efficacy at −20 °C under different protected and unprotected conditions for a maximum exposure time of 2 h for protected conditions. It is a simple and effective method to eliminate bed bugs from infested objects, linen, or furniture, but a house treatment is still necessary for a complete treatment. These are eco-friendly methods that eliminate bed bugs just by thermal shock. They are beneficial, in particular in locations with limited accessibility. In other investigations by Barbarin et al. [85] and Ulrich et al. [86], the authors tested entomopathogenic fungal strains of *Beauveria bassiana* and *Metarhizium anisopliae* in killing bed bugs. Nevertheless, its efficacy depends on humidity, making field application difficult. Besides these methods, pheromones coupled with insecticides or entomopathogens (e.g., pathogenic bacteria, fungi, etc.) [85], Ozone [87], Diatomaceous Earth [88], glue traps [89], Electromagnetic/Ultrasonic waves [90] or biological controls that involve natural predators and parasites (i.g., some arachnids such as spiders, solifuges, some mites, some bacteria of the genus *Serratia* or fungi such as *Aspergillus flavus*) [2,91] are other ways less frequently used for control.

## 7. Bed Bugs: Future Treatment

Bed bugs are a long-standing problem that made a rapid resurgence worldwide. Although they are not confirmed biological vectors of human pathogens, their impact on public health is undeniable. The keystone pressure of bed bugs as a newly emerged problem is on the health network system. It includes direct (e.g., clinical and psychological disorders) and indirect (e.g., cost of hospitalization) meta-expenses, including prevention, diagnosis, treatment, and control. In an investigation performed on the economic impact of bed bugs in a hospital in Cleveland, OH, the costs were between $125 and $1050 per infestation, with total expenses for the year of $55,915 for the hospital with an infestation rate of 1 every 2.2 days [92]. Beyond these direct costs and legal fees, they spent additional charges for active surveillance and prevention programs. The presence of patients with bed bug infestation in clinical settings demands a multidisciplinary approach involving social workers, nurses, hospital infection control personnel, housekeeping staff, and county or state public health authorities to protect other patients and frontline health care providers from bed bug infestation [23]. These costly management fees impose the additional financial implications associated with bed bugs’ presence in the health care system [21,93]. These situations generate the opportunity to discuss the ethical and moral consequences of refusing to care for patients with bed bugs and how hospitals can manage the ethics of patient autonomy and hospital safety with a reasonable policy [94]. Bed bugs can negatively affect a patient’s access to timely and appropriate health care. Because of the costs associated with decontaminating a facility infested with bed bugs, careful screening at triage should be undertaken. On the other side, identifying clinical manifestations due to bed bug bites can be tricky for physicians unfamiliar with these ectoparasites and their clinical disorders.

Furthermore, clinical symptoms may be confused with other causatives that often share similar clinical manifestations. We presented a range of clinical manifestations caused by bed bug bites and entomo-epidemiological criteria beneficial for detecting and controlling these ectoparasites (Table 1 and Table 2). Despite the growing prevalence of bed bugs, many people report little or no knowledge of them. This can even worsen among healthcare workers. In a survey performed in two hospitals in Pakistan, over one-third of nurses could not identify a bed bug [95]. This poor knowledge of bed bugs and preventive practices resulted in bed bug infestation in 72.1% of the wards [95]. Although the reasons for this lack of health awareness were not investigated, it is commonly related to the underestimation of bed bugs infestation by patients or health authorities. In another investigation performed in 2018 in student dormitories infested with bed bugs in Iran, 25 out of 100 rooms were infested, but more than 80% of students didn’t have information about the bed bugs’ biology [96]. Furthermore, infestation is considered a social shame in some populations, especially in traditional societies.

The infestation is usually investigated after clinical suspicion of bed bug bites. Because bites are an unreliable indicator of an infestation (some people don’t exhibit the symptoms), other specific bed bug evidence is needed to look for. The presence of bed bug fecal spots or exoskeletons on the mattress and bedding is a sign of bed bug infestation. Bed bugs hide commonly in small and dark places, such as household cracks, crevices, bedclothes, mattresses, bed frames, under baseboard spaces, loose or peeling wallpaper, electrical switch plates, or cable channels (Figure 2). Furthermore, they can move from an infested site to a new location by human mediation, e.g., luggage, furniture, bedding, clothing, and goods [75]. Therefore, preventing people from purchasing second-hand furniture or accurate inspection before buying, sealing cracks and crevices to discourage bed bug harborage, regularly inspecting furniture, etc., are practical actions for preventing bed bug infestations. Checking a hotel room before sleeping and examining a patient’s belongings before hospitalization is examples of such measures that can reduce the infestation risk.

According to entomo-epidemiological and clinical criteria, the treatment can rely on two main axes upon confirmation of bed bug bites. They include clinical and psychological treatments of the patients and eradication of the infestation via control management strategies. Furthermore, all in-access control methods should be evaluated based on qualitative (accessibility, simplicity, etc.) and quantitative (efficacy, expense, etc.) criteria. Proper identification of the bed bugs species, education of patients, other dwelling occupants, and landlord, a thorough inspection of both infested and other nearby areas, implementation of chemical and non-chemical control measures, and regular follow-up to ensure control of the infestation are additional steps to pursue [15]. The future resolutions can be included the following steps: (i) To gain a better knowledge of the current bed bug species/populations, their transmission way (active or passive), behavior, genetic, and resistance mechanisms to insecticides, (ii) Appropriate education (including the training for three groups of the public, PMPs (pest management professionals) and public health authorities), (iii) Application of non-chemical methods to remove current infestations and the strategies to prevent future infestations and finally (iv) Development of new chemical and non-chemical techniques by improving the formulation of currently existing insecticides.

## 8. Conclusions

Bed bugs are responsible for several disorders in humans affecting daily life. Although they are not known as biological vectors of pathogens, they cause clinical, psychological, and economic consequences. Despite the resemblance in clinical symptoms exhibited by patients, their biting can be discriminated from other hematophagous ectoparasites according to entomo-clinical criteria.

Bed bug management continues to be a significant challenge for many communities. The emergence of bed bug populations resistant to insecticides can aggravate this situation. Nowadays, several chemical and non-chemical methods are reported in the literature, but they are mostly inefficient or effective in specific conditions or on a limited scale. Therefore, control management strategies for bed bugs encompass a multidisciplinary approach utilizing nonchemical and chemical methods and concentrate on four priorities, (i) prevention, (ii) surveillance and Integrated Pest Management, (iii) education and communication, and (iv) research of new high throughput methodologies or molecules effective for bed bug’s eradication.

## Figures and Tables

**Table 1:** Diverse criteria of bed bugs detection based on visual inspection.

Infestation Scale	Bed Bug’s Bite	Black Spot (Excretion)				Bed Bugs’ Presence *				Visibility during the Day			
		No	Yes			No	Bed **	Other Parts of the House	Neighbor Locations	No	Yes		
			Low	Moderate	High						Low	Moderate	High
0	X	√				√				√			
1	√		√				√			√			
2	√			√			√	√		√			
3	√				√		√	√			√		
4	√				√		√	√				√	
5	√				√				√				√

*: Presence of adults, eggs, or nymphal stages; **: including bed, mattress, and the area around the bed.

**Table 2:** Discriminative entomo-clinical criteria of hematophagous arthropods bites.

Hematophagous Arthropod	Entomological Criteria									Clinical Criteria						
	Sex	Stage			Bloodfeeding Way		Bloodfeeding Time			Bite Feeling		Bite Location		Bite Spot Pattern		Manifestations
		Adult	Nymph	Larva	Solenophagy	Telmophagy	Sunset and Night	Diurnal	Any Time	Painful	Painless	Exposed Area	All body	Sporadic	Clustered	
Bed Bug	♀♂	√	√		√		√				√	√			√	Zigzag or straight pattern (see Figure 1 and Figure 3)
Mosquito	♀	√			√		√				√	√		√		Red itchy papules
Biting Midge, Sandfly	♀	√				√	√			√		√		√		Reddish swollen bump
Louse	♀♂	√	√		√				√	√			√ *		√	Small blue or red spot with inflammation and irritation
Tick	♀♂	√	√	√		√			√		√	√		√		Reddish volcanic papule with central hole
Flea	♀♂	√				√			√	√			√ **		√	Small reddish macule, papule, or nodule
Tsetse Fly	♀♂	√				√		√		√		√		√		Reddish sore (chancre), itchy boil-like swelling
Horse Fly, Black Fly	♀	√				√		√		√		√		√		Papules, vesicules, pruritus and erythematus weals

*: depending on the lice species; **: particularly lower extremities.

## Data Availability

Not applicable.
